# Stepwise reduction of graphene oxide and studies on defect-controlled physical properties

**DOI:** 10.1038/s41598-023-51040-0

**Published:** 2024-01-02

**Authors:** Poulomi Das, Sk Ibrahim, Koushik Chakraborty, Surajit Ghosh, Tanusri Pal

**Affiliations:** 1 Department of Physics, Midnapore College, Midnapore, WB 721101 India; 2 Department of Physics, Vidyasagar University, Midnapore, WB 721102 India

**Keywords:** Materials science, Nanoscience and technology, Physics

## Abstract

Graphene oxide (GO) is a monolayer of oxidized graphene which is a convenient and potential candidate in a wide range of fields of applications like electronics, photonics, optoelectronics, energy storage, catalysis, chemical sensors, and many others. GO is often composed of various oxygen-containing groups such as hydroxyl, carboxyl, and epoxy. One appealing method for achieving graphene-like behavior with sp^2^ hybridized carbon is the reduction of GO *i.e.* formation of reduced graphene oxide (RGO). A stepwise reduction GO to form a family of RGO, containing various quantities of oxygen-related defects was carried out. Herein, the defects related chemical and physical properties of GO and the RGO family were studied and reported in an effort to understand how the properties of RGO vary with the reduction rate. Although there are several reports on various features and applications of GO and RGO but a systematic investigation of the variation of the physical and chemical properties in RGO with the varying quantities of oxygeneous defects is imperative for the engineered physical properties in achieving the desired field of applications. We have attempted to look at the role of sp^2^ and sp^3^ carbon fractions, which are present in RGO-based systems, and how they affect the electrical, optoelectronic, and adsorption characteristics.

## Introduction

Graphene, a 2-D atomically thin honeycombed structure of carbon has attracted much attention due to its remarkable electronic and optical properties^1,2^. The distinctive optical, mechanical, and electrical features of graphene and graphene-based materials make them desirable for a wide range of applications^3–5^. The inability to synthesize graphene in large numbers with excellent purity is one of its key disadvantages. Simply speaking, graphite is made up of multiple layers of graphene. A well-known method for producing graphene-based materials involves oxidizing graphite powder to produce graphene oxide (GO), which is then chemically reduced to produce reduced graphene oxide (RGO). Hydrazine hydrate, sodium borohydride, sodium hydroxide, sodium dithionite, L-ascorbic acid, and formaldehyde^6–8^ all have been employed as reducing agents to produce RGO. RGO represents a class of materials with various physical/chemical properties that vary greatly upon various sources of oxygen-containing groups, including hydroxyl, carboxyl, and epoxy as well as their amount^9^. The forces between the individual graphene layers can be modified by this method, which can then be exfoliated to reveal the individual graphene oxide (GO) layers. The completely oxidized graphene exhibits a higher band gap of around 2.2 eV, after simple reduction that can be lowered to a few electron-volt by simple reduction^10^. GO is made of the assembly of sp^2^ and sp^3^- bonded atoms, whereas an ideal graphene contains 100% sp^2^-hybridized carbon atoms and has zero band gap. The structural and electronic behaviors of GO can be tuned by controlling the ratio of sp^2^ and sp^3^ hybridized carbon. Structurally, GO is similar to a honeycombed structured graphene sheet with its basal plane having oxygen-containing groups such as hydroxyl, epoxy, and carbonyl groups, attached to the edges. Chemical and thermal reduction are the two most frequently used methods to reduce GO. In the thermal reduction method although the final material may have outstanding barrier properties but it requires high temperatures, and the resulting RGO can be a brittle material. Chemical reduction is the process of combining GO with a reducing agent, such as hydrazine, hydrazine hydrate, sodium borohydride, ascorbic acid, and many others. Chemical reduction has the major benefit of being relatively simple to scale up to industrial manufacturing. RGO offers exciting electronic, chemical, and mechanical properties that are currently being investigated for advanced electronics, optoelectronics, and energy storage applications^11–13^. People have already studied the photo response properties of solution-processable RGO devices either for UV or IR light detection^14,15^. In addition, due to the large surface area, RGO may considered to be an ideal template for adsorbent towards the elimination of dyes and organic contaminants from the aquatic environment^16^. The effectiveness of the RGO-based composite towards the efficient removal of organic water pollutants is strongly related to the pH of the medium, porosity and the surface area of the adsorbent, nature of dyes, temperature, ability to interact with dye molecules, etc.^17–20^. The presence of a large no of adsorption sites, mainly negatively charged oxygeneous defects, wrinkled surface morphology, and π-electron rich domains on the mat-like planner configuration of the RGO makes it favorable for the adsorption of cationic dyes. RGO is a representative of a class of materials with various physical and chemical characteristics and emerged as a new member of carbon-based nanoscale material that is considered a prospective source of “cheap graphene”^21^. Its property is strongly related to the reduction time, reducing agents, and different other factors. The effect of the reduction time of GO on the dielectric characteristics of the RGO-BaTiO_3_ composite was studied by Jun et al.^22^. The infrared sensitivity of thermally and chemically reduced GO thin films was studied by Al-Hamry et al.^23^. Li et al.^24^ studied the reduction time effect of GO on the structure and properties of RGO. Step-by-step reduction of GO and its impacts on colloidal, chemical^25^ and photoluminescence properties^26,27^ were also investigated systematically. Several articles have been published on various features and applications of GO and RGO^11–13,28–30^; however, systematic research is highly required to get insight into the effect of various oxygen levels on the physical and chemical properties of RGO.

In our present study, we have tried to investigate the role of sp^2^ and sp^3^ carbon fractions, present in RGO-based systems towards controlling the electronic, optoelectronic, and adsorption properties. Herein, the defect-controlled photocurrent generation, and adsorption properties of RGO composites with varying sp^2^ contain are studied extensively. The carbon sp^2^ fraction can be tuned simply by varying reduction times using hydrazine hydrate as a reducing agent. The effect of reduction on the photocurrent generation in GO/RGO-based large area thin film photodetector has also been reported.

## Experimental section

### Chemicals

Graphite powder, Potassium Persulfate [K_2_S_2_O_8_], Phosphorus Pentoxide [P_2_O_5_], Sodium Nitrate [NaNO_3_], Potassium Permanganate [KMnO_4_], Hydrazine Hydrate [NH_2_NH_2_.H_2_O], Potassium Bromide [KBr] and Rhodamine B (RhB) dye of Sigma-Aldrich make and acid like H_2_SO_4_, HCl and Hydrogen Peroxide [H_2_O_2_] of Merck makes were used in the present study.

### Material synthesis

**Figure 1:**
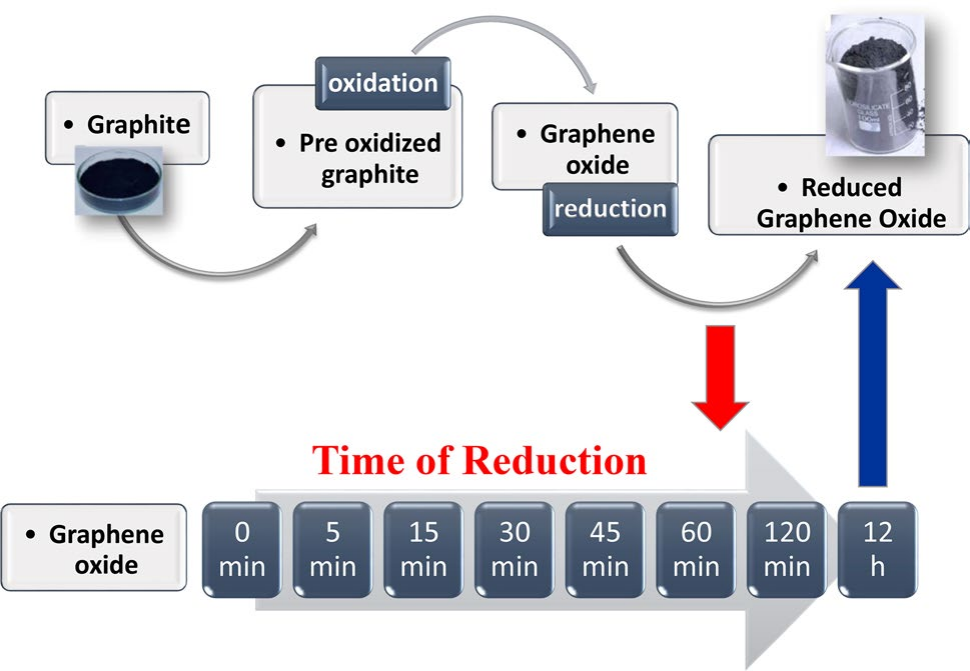
Synthesis scheme of GO and RGO of different time of reduction.

Modified Hummers’ method was employed to synthesize GO from natural graphite powder as a precursor^9,31,32^. In a brief, graphite powder (2 g), K_2_S_2_O_8_ (1 g), and P_2_O_5_ (1 g) were placed in a round bottom flask and 20 mL of 98% H_2_SO_4_ was added to the mixture that had been prepared earlier. The mixture was stirred continuously for six hours at 80° C in an oil bath and pre-oxidized graphite was prepared. This pre-oxidized graphite was then washed in double distilled water (DDW) and dried at 60 °C. In the oxidation step, 0.2 g pre-oxidized graphite powder along with 0.1 g NaNO_3_ were kept in a round bottom flask of 100 mL capacity. In the next step concentrated H_2_SO_4_ (5 mL) was added to the mixture and kept in an ice bath to maintain the temperature of the solution below 20 °C. KMnO_4_ (0.6 g) was added to the suspension step by step, and transferred into a second bath, and kept for 3 h. The bath temperature was maintained at 35 °C. 10 mL DDW was then added slowly which produced a large exotherm. To keep the reaction temperature at 80 °C, external heating was applied for 1 h. Further 30 mL of warm water was added to dilute the suspension and it was allowed to cool naturally. At room temperature, 12 mL of H_2_O_2_ (3 weight percent) was added to terminate the reaction. Consequently, the GO suspension was produced and stirred for a duration of 12 h. To eliminate the metallic ions, an aqueous solution [H_2_O: HCl = 10:1] was added and kept for 5 h under stirring conditions. After that, the GO solids were rinsed with DDW to keep the pH at 6 and dried up at 60 °C. Exfoliation was carried out by sonication of 20 mg of GO, dispersed in 20 mL of DDW for 30–40 min. Finally, RGO was formed by the reduction of GO. The reduction process was performed by the addition of Hydrazine hydrate (1μL/1 mg GO) as the reducing agent. After that, the mixture was heated at 80 °C while being stirred for 5, 15, 30, 45, 60, and 120 min to generate RGO sheets with varying levels of reduction efficiency. Other samples were left in hydrazine for 12 h. The colour of the dispersion changes to black and segregated after reduction which was thoroughly dispersed brown-coloured before the reduction. All the samples were collected by centrifugation and dried under vacuum. The graphical presentation of the GO synthesis route and reduction steps are shown in Fig. 1.

### Characterization

The synthesized GO and RGO Powder samples were characterized by X-ray diffractometer (XRD, Rigaku Miniflex 600, with Cu Kα radiation, λ = 1.5406 Å), and the diffraction patterns were recorded over 2θ range of 3°–50° with an operating voltage of 40 kV. The surface micromorphology of as-synthesized GO and RGO was studied with a scanning electron microscope (SEM; Zeiss Merlin). The Raman spectra of GO and RGOs of different reduction times were collected at room temperature by a Triple Raman Spectrometer (Renishaw, inVia Raman Microscope attached with 532 nm excitation laser) to evaluate the defect states present in the samples as well as to calculate the reduction efficiency from the carbon sp^2^ percentage. To ascertain the structure and functional groups present in GO, infrared transmittance spectra were recorded on a Fourier Transform Infrared (FTIR) spectrometer (Perkin Elmer—Spectrum 100) using a KBr pellet. Ultra Violet-Visible (UV–vis) absorption spectra of the samples dispersed in the aqueous medium were collected on a spectrophotometer (Agilent Cary Series). Room temperature steady-state photoluminescence (PL) study was carried out on a spectrofluorometer (Perkin Elmer LS 55). An Autosorb iQ-MP instrument (Quanta chrome instrument) was employed to measure the specific surface area and the pore volume by the Brunauer–Emmett–Teller (BET) method using N_2_ adsorption isotherms.

### Device fabrication and optoelectronic transport properties study

Two terminal thin film photodetectors were fabricated on a pre-cleaned glass substrate by drop-casting stable suspensions containing GO and RGO of different reduction times. The solvent was allowed to evaporate for an hour. A pair of parallel electrodes was drawn using conducting silver paint (Ted Pella). Devices were annealed at 60 °C for better contact between the films and metal electrodes as well as better contact between individual sheets. The electrical properties under dark and illumination were investigated in a probe station connected to a Keithley 2611A source meter in ambient conditions using the standard two-probe method. Data were collected using LabTracer2.0 interfaced with the data acquisition card. A solar light simulator (Newport) was used as an illuminating light source. The incident illuminated light power was measured with an optical power meter (Newport). The dark current (I_dark_) was subtracted from the current under light illumination (I) to determine the photocurrent (I_L_) [I_L_ = I − I_dark_].

### Evaluation of adsorption capacity

Generally, 50 mg of adsorbent (GO or RGO) was added to 100 mL of aqueous solution of Rhodamine B (RhB) under stirring conditions. The adsorption capacity of RhB by the adsorbent was monitored through a UV–visible spectrophotometer. The concentration of leftover dye was estimated based on the peak intensity of RhB at 554 nm in an aqueous solution. The adsorption capacity (${{\text{Q}}}_{{\text{e}}})$ and the percentage of removal efficiency of different adsorbent was calculated by using the following equations^33,34^:

1 $$\mathrm{The \; adsorption \; capacity } \; {{\text{Q}}}_{{\text{e}}}=\left({C}_{0}-{C}_{e}\right) \times \frac{V}{W}$$

2 $$\mathrm{Percentage \; removal \; efficiency}= \left(1- \frac{{C}_{e}} {{C}_{0}} \right)\times 100\%$$

where ${{\text{Q}}}_{{\text{e}}}$ (mg.g^-1^) is the adsorption capacity at the equilibrium, ${C}_{0}$ (mg L^-1^), the initial concentration, ${C}_{e}$ (mg.L^-1^), the concentration at the equilibrium, V the volume solution in L and W the amount of adsorbent used as adsorbent.

## Results and discussion

**Figure 2:**
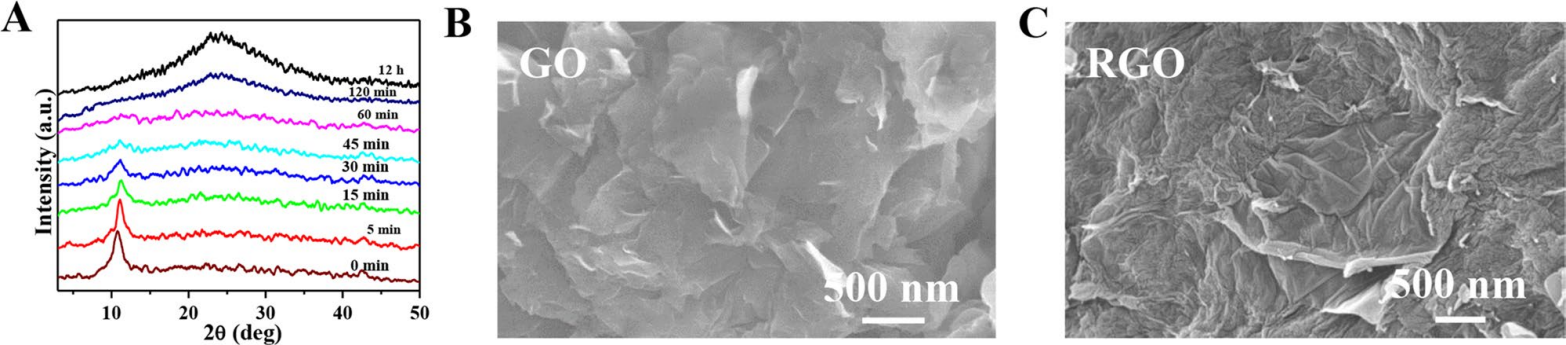
(**A**) XRD pattern of the powder sample of GO and RGO with different reduction times. SEM image of (**B**) GO and (**C**) RGO-12 h of reduction.

X-ray diffraction (XRD) pattern of the powder sample of GO and RGO with different reduction times is compared in Fig. 2A. An intense sharp peak centered at 10.51° corresponding to the inter-planar spacing (*d*-spacing) 0.841 nm, assigned to the (100) reflection plane is observed in the XRD pattern of GO^35^. This value is much larger than that of *d*-spacing of natural graphite (0.334 nm) due to the presence of oxygenated functional groups and intercalated water molecules inside the interlayer galleries of hydrophilic GO. A gradual shifting of the distinct peak to a broadened hump centered at 2θ ~ 23° is observed after the reduction of GO with different reduction times. No significant features of crystallinity are observed for the sample prepared under 1 h reduction. The hump near 2θ ~ 23° appeared after 120 min reduction and finally, the hump become prominent for the sample reduced for 12 h confirming the restoration of a graphene-like structure through the successful reduction of GO. The SEM image of as-synthesized GO and RGO is displayed in Fig. 2B and C, respectively. As shown in the figure, the GO exhibited folded and crumpled sheet-like shapes. These folded sheets of GO were well reduced and had an exfoliated sheet-like morphology which increased the surface area. Similar types of morphology have been seen in the literature, which suggests the successful formation of RGO by the reduction of GO^36^.

**Figure 3:**
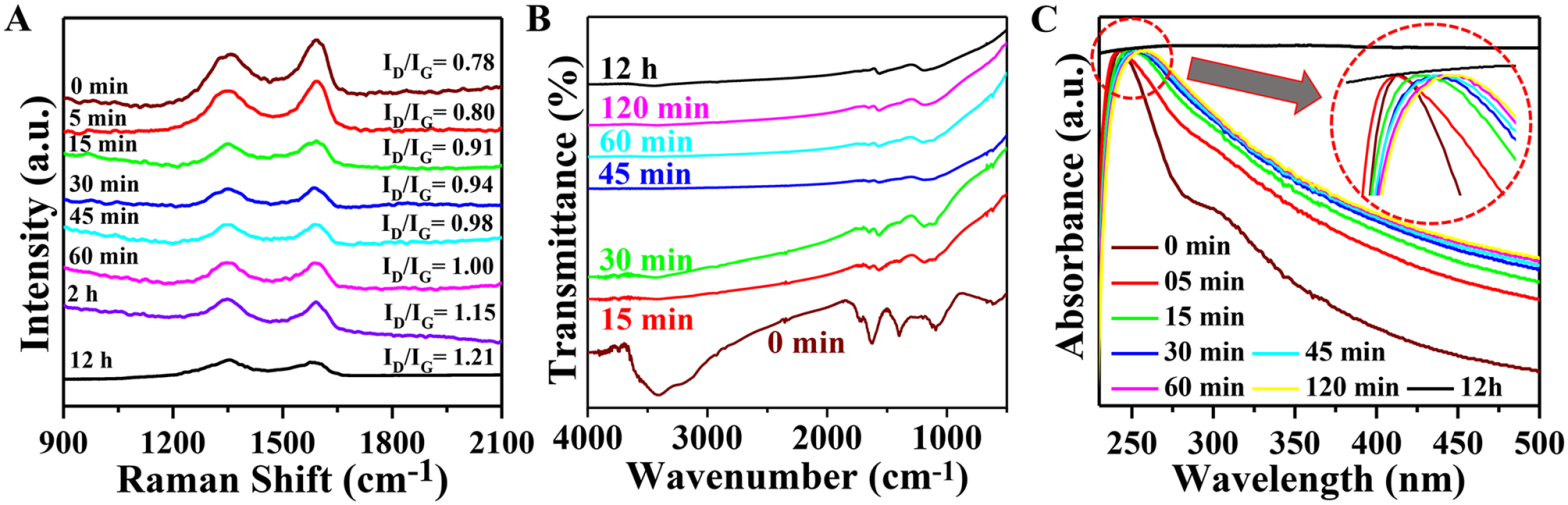
(**A**) Raman (**B**) FTIR and (**C**) Optical absorption spectrum of GO and RGO with different reduction times. The Zoomed image of Optical absorption spectrum (inset).

Raman spectroscopy measurement was employed to get the structural information of the GO and RGO sheets. The number of defects on the graphene structure can be determined by the intensity ratio of the D/G bands. The D band is caused by out-of-plane vibrations ascribed to the existence of structural flaws, whereas the G band is caused by in-plane vibrations of sp^2^-linked carbon atoms. Raman spectra for all the samples are compared in Fig. 3A. The high bandwidth and similar intensity of the significant D peak (~ 1356 cm^-1^) to the G peak (~ 1590 cm^-1^) imply structural flaws in GO^37^. As observed in Fig. 3A, the intensity ratio (I_D_/I_G_) of the bands increases gradually with the reduction time from 0.78 (as-synthesized GO) to 1.21 after 12 h reduction (RGO-12 h). It suggests that the removal of oxygen functional groups during the reduction of GO increased the crystalline size of sp^2^ carbon domains. Nonetheless, the still-significant D band intensity of RGO indicates the existence of sp^3^ carbons, residual oxygen functions, and defect sites.

By using Fourier Transform Infrared spectroscopy (FTIR), the chemical structure of GO was examined both before and after the reduction. The results are shown in Fig. 3B. FTIR spectrum of as-synthesized GO displays six dominating absorption peaks at 3409, 1730, 1623, 1394, 1098, and 611 cm^-1^ that are attributed to -OH stretching, C=O stretching, skeletal vibrations of unoxidized graphitic domains, -OH deformations of the C–OH groups, stretching vibrations of C-O (epoxy or peroxide) groups, and OH out-of-plane bending respectively^38–40^. As can be seen, the intensities of all the peaks related to oxygen-containing functional groups (epoxy and carbonyl) are drastically diminished with the increase of the reduction time. These findings demonstrate the chemical conversion of GO to RGO by the process of reduction. Markedly, a new peak appeared at 1568 cm^-1^ which is the characteristic fingerprint of skeletal vibration of the graphene domain. These spectroscopic changes reveal the stepwise successful reduction of GO with reduction time.

Figure 3C compares the UV–Vis absorption spectra of GO and RGO (of various reduction times) in an aqueous solution. As-synthesized GO has a plasmon peak around 243 nm owing to the π–π* transition of the C–C aromatic rings and a shoulder peak at 300 nm due to the n- π* transition^41^. The peak at 243 nm is gradually red-shifted towards 267 nm (inset of Fig. 3C) and the intensity of absorption beyond 240 nm is enhanced with an increase in reduction time. It is also observed the shoulder peak of GO at 300 nm markedly vanishes, signifying the restoration of a highly conjugated graphene-like structure leading to hydrazine reduction^42^. The optical band gap of GO and RGO of different time of reduction has been estimated from the Tauc relation^43,44^ and are presented in Table 1. The variation of bandgap energy with reduction time is presented as Figure S1, in Supporting Information (SI).

**Table 1:** Variation of band gap energy with the time of reduction.

Reduction time	0 min	5 min	15 min	30 min	45 min	60 min	120 min	12 h
Band gap (eV)	3.50	3.44	2.90	2.77	2.54	2.42	1.89	1.50

**Figure 4:**
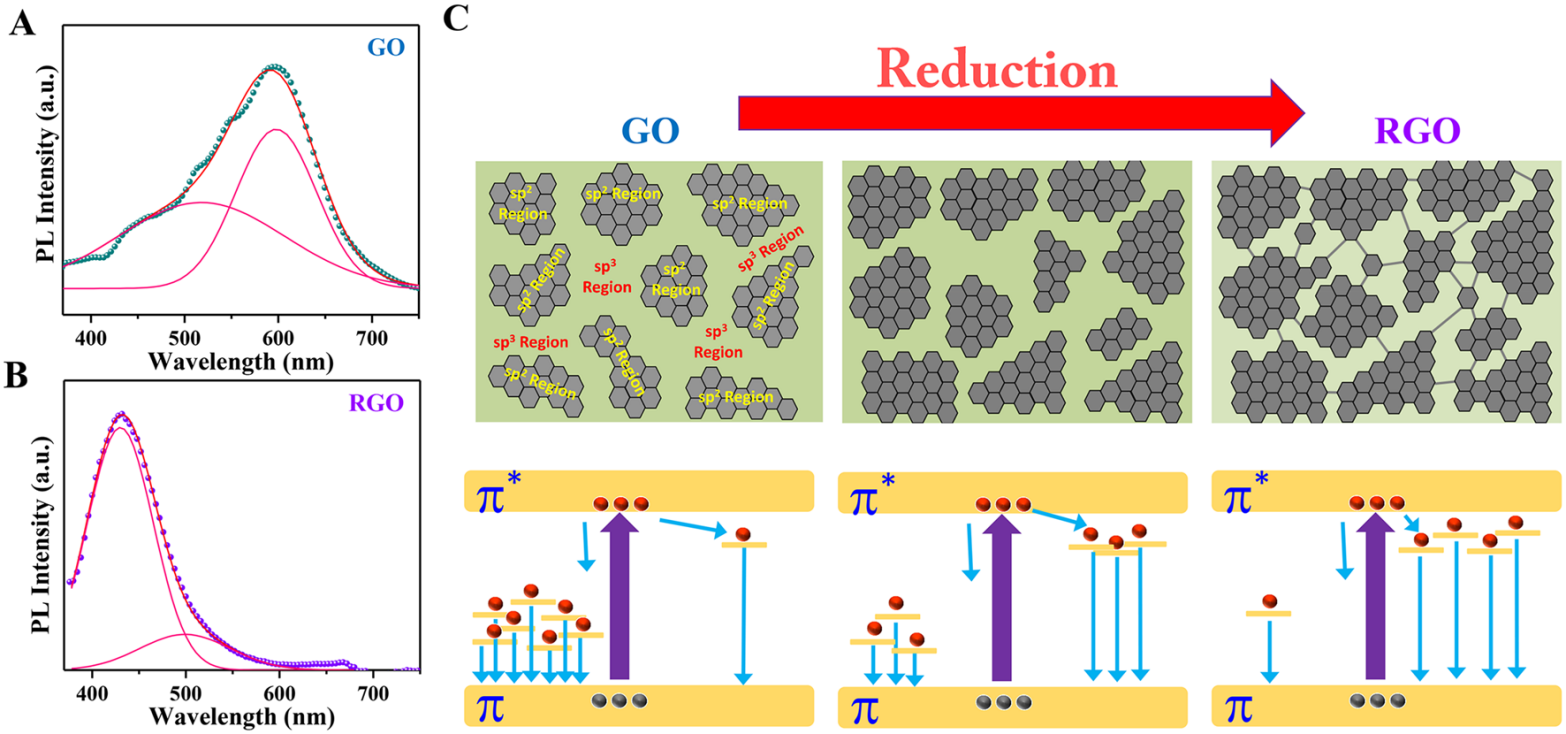
PL spectra of (**A**) GO and (**B**) RGO-12 h. (**C**) Schematic presentation of the possible mechanism of PL of GO and RGO.

The PL spectrum of as-prepared GO (Fig. 4A) reveals a broad PL response that originates from structure-related defects and spans 400–750 nm^45^. The absence of an absorption peak in the UV–Vis spectrum of GO within this particular wavelength range rules out the possibility of the band edge transition. A significant portion of the distorted C atoms in graphene oxide have oxygen-containing functional groups attached to them. The broad PL spectrum is primarily caused by charge transitions in disorder-induced localized states or bond alterations within the graphene oxide plane. The PL peak shifts towards shorter wavelengths and bandwidth becomes narrower for the RGO after 12 h reduction (Fig. 4B). The PL spectra of GO suspensions were deconvoluted into two Gaussian-like peaks with distinct wavelengths, one centered at wavelengths 600 nm and a small emission peak centered at approximately 490 nm. After 12 h of reduction, the peak in the longer wavelength region moved from 600 to 500 nm and the peak of the shorter wavelength region (490 nm for GO) shifted to 430 nm. Interestingly, the PL of GO is strongly controlled by the peak at 600 nm (structure-related defects)^45^ whereas for highly reduced GO (RGO-12 h) it is fully dominated by the peaks at 430 nm which is highly conjugated graphene-like structure (sp^2^ structure). Remarkably the intensity of the defect-related peak becomes very faint after the reduction. We calculated the percentage of the peak related to sp^2^ structure for GO and it was only 36% whereas for RGO-12 h it is as high as 88% which is also supported by the IR and Raman spectrum data.

All the characteristics study confirms that as-synthesized GO contains several oxygen-containing groups C–OH (hydroxyl), and C–O–C (epoxide). It is already established that GO consists of a mixture of sp^2^ and sp^3^ bonded C atoms^46^. Our synthesized GO and RGO systems can therefore be viewed as a heterogeneous blend of highly insulating sp^3^ and highly conducting sp^2^ functionalized sheets. A schematic of the well-distributed sp^2^ domains is demonstrated in Fig. 4C where the insulating sp^3^ domains are represented by the void green regions. In GO the sp^3^ domains dominate the entire system. The size of the sp^2^ domains can be tuned with the reduction time and it is increased with the increasing reduction time of GO by hydrazine that efficiently reduces the opening between sp^2^ regions. The conducting sp^2^ (RGO) islands are separated by insulating sp^3^ (GO) matrices. Based on the above schematic presentation we have tried to explain the PL spectrum of GO and RGO and the possible mechanism is shown in the bottom row of Fig. 4C. The blue shift of PL spectra of GO after reduction is therefore attributed to the variation of the heterogeneous electronic structures of GO and RGO with variable sp^2^ and sp^3^ hybridizations through reduction. As observed from the IR data GO contains oxygenous group, the optical transitions from these disorder-induced localized states may be primarily responsible for the prominent broad emission band centered at 600 nm in the as-prepared GO. The quantity of these disorder-induced states declines with deoxygenation by reduction, resulting in a drop in the intensity of the emission peak. In the process of reduction, some of the carbon lattices in the original distorted sp^2^ domains can form new graphitic domains of sp^2^ clusters. As a result, a highly intense emission peak in the 430 nm is observed.

**Figure 5:**
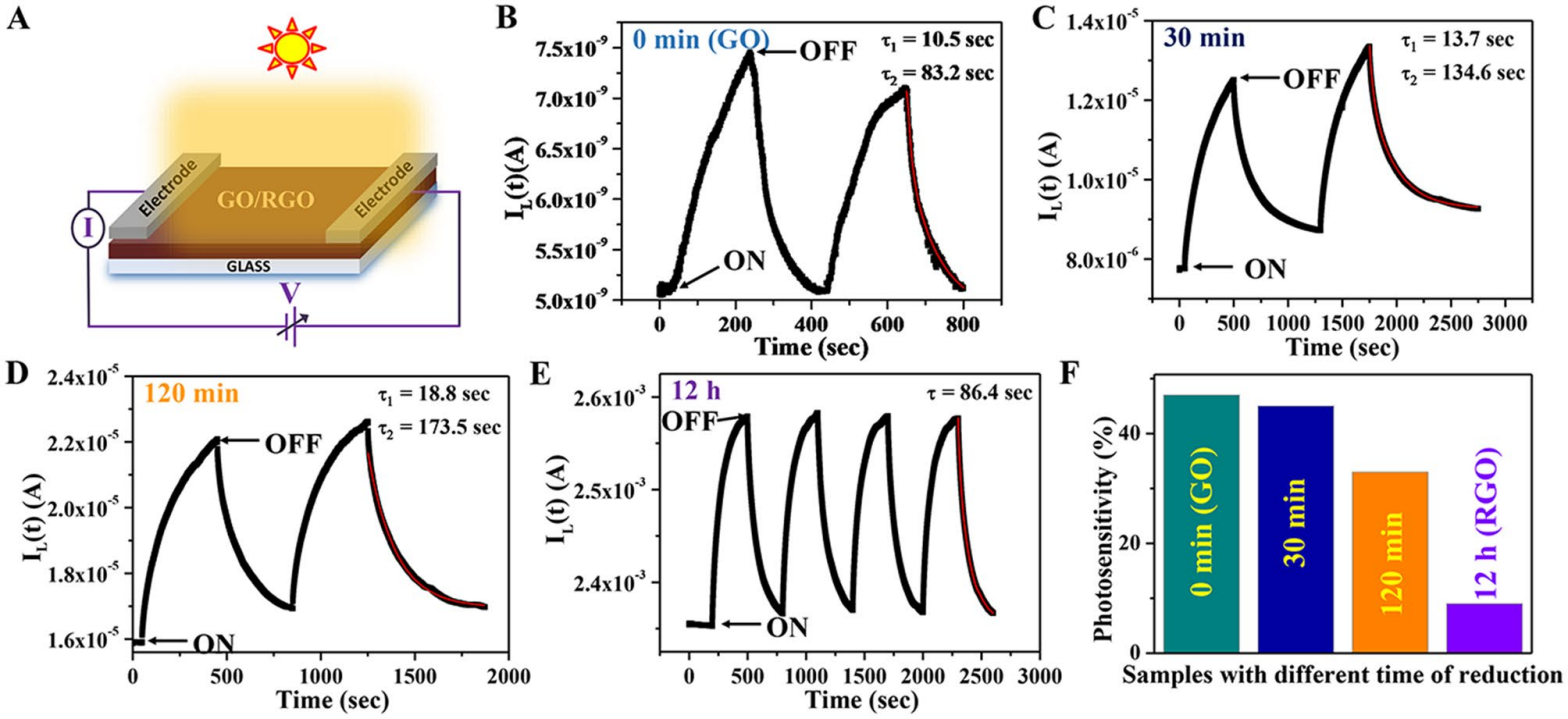
(**A**) Cartoon of our thin film photodetector device. (**B**–**E**) Dynamical photoresponse and (**F**) Comparison of photosensitivity of GO and RGO (of different reduction time) thin film device.

To investigate the influences of oxygenous groups on the visible light optoelectronic transport properties, the photo response was studied on a prototype large area thin-film photodetector based on GO and RGO (reduced time 30, 105 min, and 12 h). Figure 5A represents the schematic of the thin film photodetector device of GO/RGO with different times of reduction along with a setup for electrical measurement. All the detectors were measured in identical experimental conditions (sweeping of bias voltage from 0 to + 2, reverse + 2 V to − 2 V, and back to 0 V) both in dark and light (100 mw/cm^2^) and are presented in Fig. S2 in SI. As shown in the figure, a hysteresis loop like I–V characteristics is present in GO both in dark and light. The origin of the hysteresis loop can be attributed to the defect states present in the GO acting as trap centers and hindering the charge transportation in the GO-based device in the reverse direction. Although a very small area, a noticeable hysteresis loop-like I-V characteristics come out for the sample (RGO-30 min) indicating the existence of oxygeneuous defects that happen to be more active under the influence of visible light. Under identical experimental conditions, the other detectors (based on samples RGO-120 min and RGO-12 h) do not show either nonlinear or any hysteresis loop-like I–V behaviors. These are expected as the defect states become weaker beyond 30 min of reduction time. To further confirm the defect-controlled visible light photo response in GO-based detectors, we have studied the dynamical photoresponse of all the devices. Figure 5B–E shows how photocurrent changes over time when exposed to a simulated solar light (100mW/cm^2^) at a constant bias voltage of 2 V.

Periodically, the solar light was turned ON and OFF by the mechanical chopper at regular intervals of time. All the fabricated detectors respond very well to solar light. As shown in Fig. 5B, the GO-based composite shows 47% photosensitivity (ratio of current under illumination to the dark current). The photosensitivity decreases with the increase of reduction time and becomes only 6–8% for the RGO (12 h). When the source is turned "ON" the devices respond to the light, and when it is turned "OFF" photocurrent gradually decreases.

The dynamic response is described as I(t) = I_dark_ + B exp − (t − t_0_)/τ_1_ + C exp − (t − t_0_)/τ_2_ (decay), τ_1_ and τ_2_ are the fast and slow responses time constant; t-t_0_ is the time when the light had been turned off; I_dark_ is the current at dark condition; B and C are the fitting constants^47^. The time constant was calculated to be about 10.5 and 83.2 s for decay for GO indicating very rapid photocurrent decay initially followed by a very slow decay process. For τ_1_ and τ_2_ the devices with reduction times 30 min and 120 min are 13.7, 134.6 s and 18.8, 173.5 s respectively. The dynamic response for the RGO-12 h-based device for the same experimental condition shows an exponential behavior with I(t) = I_dark_ + A exp − (t − t_0_)/τ (decay), and τ was calculated to be about 87 s. The variation of the photosensitivity with the reduction time is shown in Fig. 5F. The photo-responsivity (R) an important figure of merits of an optical detector gives the light to electrical energy conversion efficiency. The R values are measured for all the devices which is increased by five orders of magnitude for RGO-12 h (4 mA/W) with respect to synthesized GO (4 × 10^–5^ mA/W).

**Figure 6:**
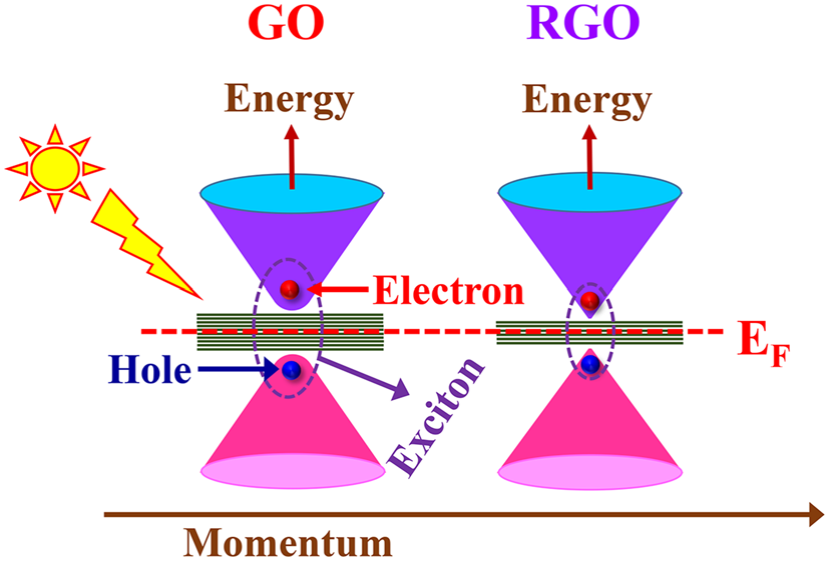
Schematic of the proposed excitonic model of photocurrent generation.

From the study of optoelectronic transport properties, it is observed that the dark current as well photocurrent of the RGO-based detector increased many fold depending upon the reduction times. We have tried to explain these features with the help of the E-k band diagram of GO and RGO which is shown in Fig. 6. Graphene's unique π-π* band structure makes it a semi-metallic substance. The conduction and valence bands are symmetrical at the Dirac point (the point of charge neutrality). Because of its inherent zero-band gap energy, graphene is not the best candidate for photodetection. Formation of GO and transforming it into RGO is a simple way to form a gap and tune the gap by controlling defects. The fully oxidized graphene (GO) behaves like an insulator. As per the literature, the band gap of GO can be tuned in a wide range from more than 3 eV down to 1 eV or below depending on the level of reduction^48–54^. Thus, the electrical conductivity may be attuned by varying the amount of oxygen in the GO-based devices. At dark, the conductivity comes from thermally generated carriers, and the carrier concentration at a particular temperature $n\left(T\right)={n}_{0} exp\frac{-{E}_{g}}{kT}$ where ${E}_{g}$ is the band gap of the semiconductor. As the band gap of RGO is less than GO, the number of carriers is more than GO for the same temperature. Also reducing the defects helps to improve the mobility of the carrier. As a result, five orders of magnitude higher dark current is observed for RGO-12 h than the as-synthesized GO. In the GO-based system, the photocurrent is produced by the formation of the exciton (bound electron–hole pair) through photon absorption and its dissociation at the metal interface into free electrons and holes. These free charge carriers are collected to the source and drain electrode by an external voltage before they recombine. In addition, defects in the individual nanosheets and nanosheet junctions of the GO-based thin film also produce local polarization and may support the dissociation of photogenerated electron–hole pairs into free carriers. The photocurrent density depends on several parameters: the amount of light absorption and exciton formation, exciton diffusion length, and finally the amount of charge carriers and carrier mobility. Apart from this the photogenerated carriers may become trapped by the defect states that are present in the bandgap as well as at the interface of the GO (RGO)-electrode interface while drifting across the channel space. Noticeable hysteresis loop-like I–V characteristics and double exponential decaying of dynamical photocurrent of GO and RGO-30 min confirm that oxygeneuous defects are responsible for their behavior. After reduction, the device shows linear I–V as well the dynamical photocurrent follows only exponential variation which means the system is more ordered.

**Figure 7:**
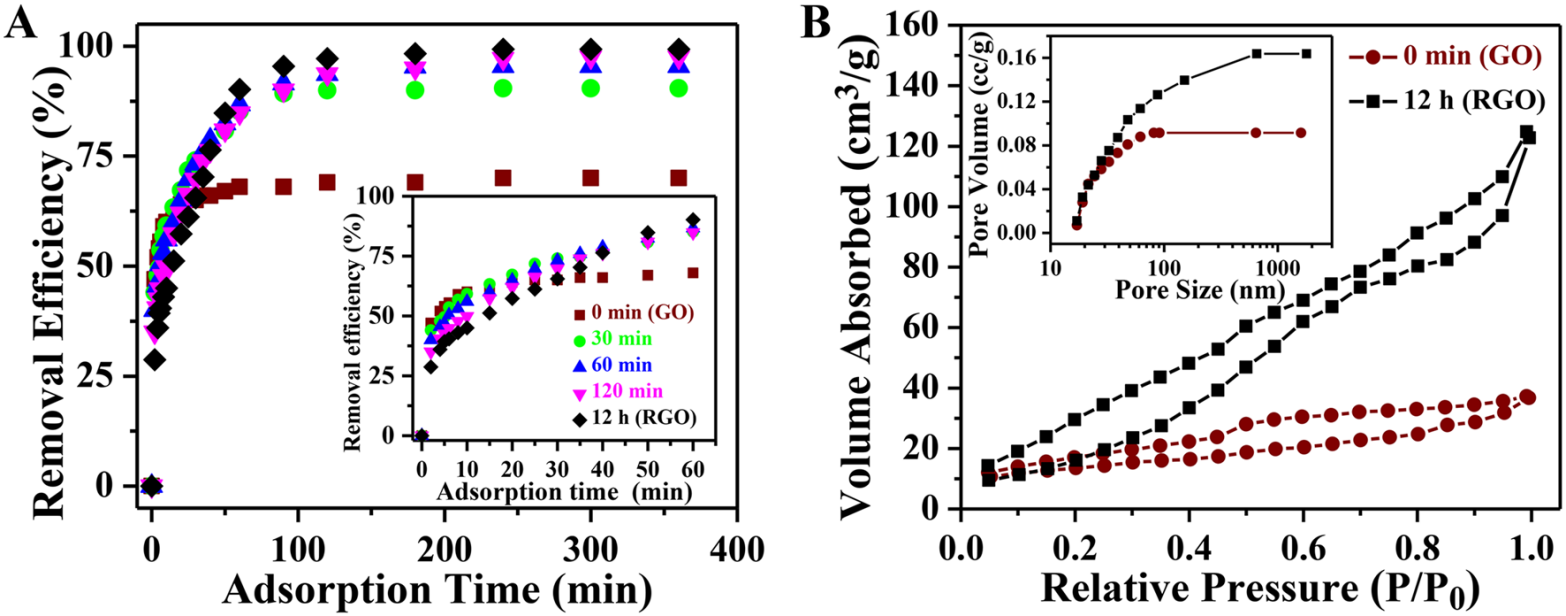
(**A**) Variation of RhB removal efficiency with illumination time of GO and RGO of different time of reduction. Zoomed view of removal efficiency (inset). (**B**) Nitrogen adsorption–desorption isotherms and the inset is corresponding pore-volume distribution curves of GO and RGO.

The large surface area and 2-D structure of GO/RGO are thought to make it an outstanding adsorbent for removing aquatic pollutants from the environment. The presence of a large no of adsorption sites; mainly negatively charged oxygeneous defects, structural defects, wrinkles, and π-electron domains in the planner structure of the RGO mat makes it favorable for the adsorption of cationic dyes^16,17^. To understand the role of oxygeneous defects present in GO on the removal of cationic dyes like RhB we have studied the adsorption properties of GO and RGO sheets. A comparative study of adsorption of RhB dye with time (t) (adsorbent amount 0.5 gm, the concentration of RhB is 200 mgL^-1^) for all the samples under identical experimental conditions was performed and presented in Fig. 7. Initially GO shows a very fast removable efficiency than other samples. As shown in the inset of Fig. 7, GO shows 47% removable efficiency in 2 min. Other RGOs RGO-30 min, RGO-60 min, RGO-120 min, and RGO-12 h show removable efficiency 44%, 40%, 35%. and 28%, respectively in 2 min. However, the RGO-12 h sample shows steady adsorption with time and ultimately it reaches a higher adsorption efficiency compared to other members. Because the GO contains a large number of negatively charged oxygeneous groups such as carboxylic, phenolic, etc., it exhibits a special attraction for cationic dyes like RhB. As the amount of oxygenous groups reduces with the increase of reduction time the initial removable efficiency decreases compared to the as-synthesized GO. After 10 min the adsorption efficiency of GO saturated and shows 62.5%. Whereas in the same conditions, the adsorption capacity gradually increases from GO to RGO with different intermediate samples (different reduction times) and RGO-12 h shows the highest adsorption efficiency which is 99% after 100 min. A large surface area along with different voids present in RGO-12 h provides a sufficient opening for the dye molecules to diffuse and become absorbed throughout the materials. RGO-12 h are considered enormous sources of π-electrons cloud. On the other hand, in an aqueous solution, cationic dye RhB will form as positively charged ions. The electrostatic, as well as the π–π interactions among the graphitic skeleton and the aromatic rings of RhB molecules, collectively facilitate RGO-12 h as a potential adsorbent towards the adsorption of RhB.

N_2_ adsorption–desorption measurements were carried out to analyze the specific surface area and pore size of GO (0 min) and RGO (12 h) samples. The corresponding isotherms presented in Fig. 7B which exhibit type IV, indicate the existence of mesopores. The corresponding pore size distribution is also shown in the inset of Fig. 7B. In GO, the space between different layers is filled with several functional groups and as a result, the isotherm of RGO is lying above that of GO^55^. We have calculated the surface area and pore volume of both GO (0 min) and RGO (12 h) by BJH (Barrett-Joyner-Halenda) method. Our results depict that the surface area of RGO (12 h) increased to 90 m^2^/g, which is more than three times higher than that of GO (29 m^2^/g). In addition to that, the pore volume was also increased from 0.037 to 0.164 cc/g after 12 h reduction. The cumulative effect of increasing surface area and pore volume facilitates efficient adsorption of RhB by the RGO^56^.

## Conclusions

GO was synthesized by modified Hummer’s method. The reduction of GO was done by hydrazine hydrate at different times. The synthesized materials were characterized both structurally and optically. The amount of reduction experienced or the reduction efficiency during the conversion from GO to RGO was confirmed by XRD, Raman, and FTIR analysis. The PL spectrum of the as-prepared GO shows a broad PL response between 400 and 750 nm originating from both the graphitic structure as well as structural defects present in it. The PL spectrum of RGO-12 h is firmly connected with the highly conjugated graphene-like structure as observed in the UV–Vis study. The possible mechanism involved in the PL spectrum has also been articulated here. The optoelectronic transport property of the GO and RGO films is strongly influenced by the oxygenous defects present in GO under solar light illumination. The photosensitivity of the detector decreases with the reduction time whereas responsivity shows reverse behaviour. Due to the high concentration of negatively charged oxygeneous groups present in the GO, it has a great affinity for RhB (catatonic dye) helps to adsorb RhB very quickly but gets statured rapidly. A large surface area along with different voids present in RGO-12 h provides sufficient opening for the dye molecules to get adsorbed throughout the materials. In addition to that RGO-12 h is considered as an enormous source of π-electrons cloud. In addition to that RGO-12 h is considered an enormous source of π-electrons cloud that supports RGO-12 h as a potential adsorbent for RhB due to the π- π and electrostatic interaction among the aromatic rings of RhB molecules and the graphitic skeleton. Thus, by adjusting the bandgap of GO with the help of controlled reduction, one may adjust its photophysical properties like optical absorption, photoluminescence, photoconductivity, and electrical conductivity, among other semiconducting material properties. This provides a foundation for a range of photonic and electronic applications. Aside from semiconductor applications, the knowledge gained about the characteristics of oxygen-containing functional groups would be helpful because functionalized graphene-based materials are now being researched in a wide range of fields. This systematic study be a very much imperative for the engineered physical properties of the GO and RGO in achieving the desired field of applications.

### Supplementary Information

Supplementary Figures.

## Data Availability

The datasets used and/or analyzed during the current study are available from the corresponding author upon reasonable request.
