# Supplementary material for: Stepwise reduction of graphene oxide and studies on defect-controlled physical properties
Source: Sci Rep. 2024 Jan 2;14:294. doi: 10.1038/s41598-023-51040-0 (PMC10762075; doi:10.1038/s41598-023-51040-0)
Supplement: Supplementary file 1 — Supplementary Figures. [file 41598_2023_51040_MOESM1_ESM.docx]

### ***Supporting Information for***

**Stepwise reduction of graphene oxide and studies on defect-controlled physical properties**

Poulomi Das^1^, Sk Ibrahim^2^, Koushik Chakraborty^2^, Surajit Ghosh^2,*^, Tanusri Pal^1,*^

^1^Department of Physics, Midnapore College, Midnapore 721101, WB, India

^2^Department of Physics, Vidyasagar University, Midnapore 721102, WB, India

^*^Corresponding authors: ^1^tanusripal@midnaporecollege.ac.in (TP);

^2^surajit@mail.vidyasagar.ac.in (SG);

**
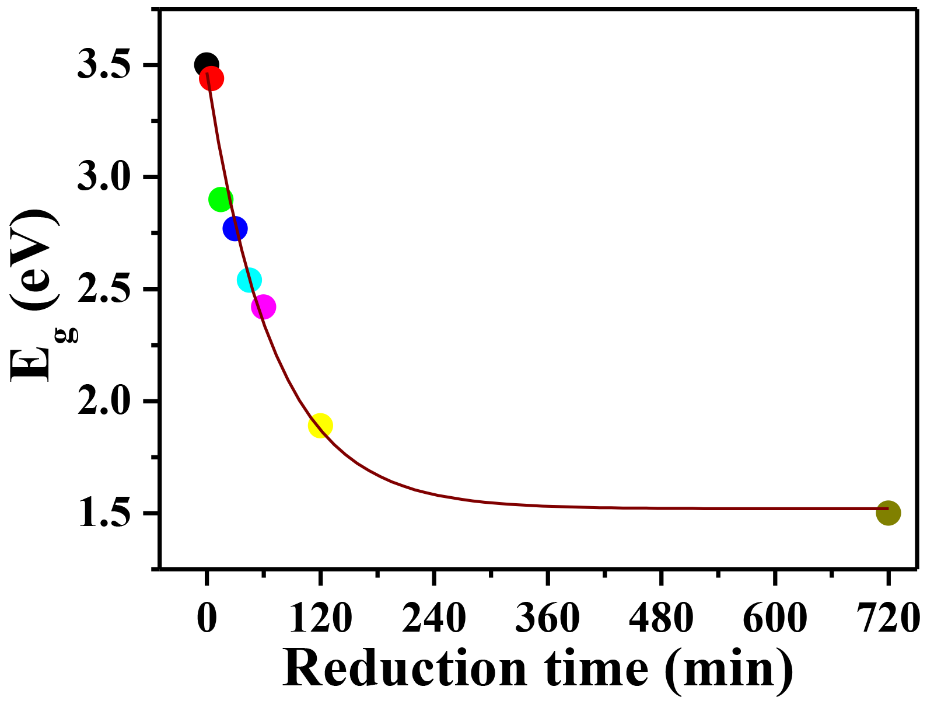
**

Figure S1 The variation of band-gap energy with reduction time. The solid line is a guide to the eye.

**
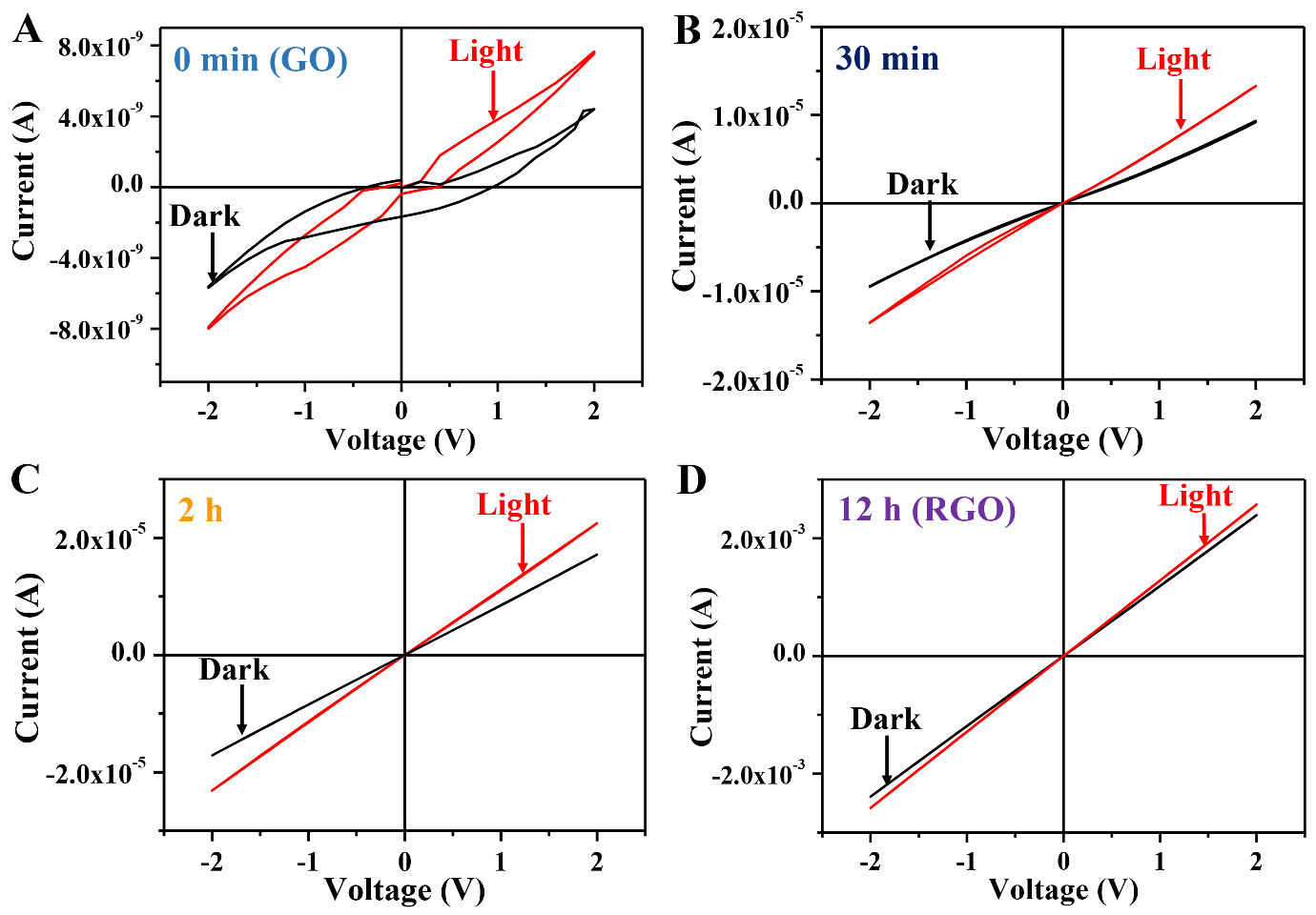
**

Figure S2 (A-D) Full cycle I-V characteristics of GO and RGO (of different reduction time) thin film devise under dark and illuminated conditions.
